# Global overview of anterior cruciate ligament reconstruction in children and adolescents over the past 20 years: a bibliometric analysis

**DOI:** 10.1186/s13018-024-04829-2

**Published:** 2024-06-13

**Authors:** Jiamin Liang, Yuxin Luo, Yingzhen Yang, Huanyu Xie, Zirong Huang, Mingjin Zhong, Weimin Zhu

**Affiliations:** 1 Department of Medicine, Shenzhen University, Shenzhen, Guangdong Province China; 2 Department of Sports Medicine, The Second People’s Hospital of Shenzhen City, The First Affiliated Hospital of Shenzhen University, Shenzhen, Guangdong Province China

**Keywords:** Bibliometrics, Web of science, Children, Adolescents, Anterior cruciate ligament

## Abstract

**Objectives:**

The objectives of this paper is to conduct a bibliometric analysis to examine the research status and development trend of anterior cruciate ligament injury and reconstruction in children and adolescents over the past 20 years.

**Design:**

Descriptive Research.

**Methods:**

This study obtained information regarding studies on Anterior Cruciate Ligament Reconstruction in Children and Adolescents from the Web of Science Core Collection database. Visual and bibliometric analysis were conducted using VOSviewer, Origin 2022, Pajek64 5.18and Excel 2019. These analytic tools facilitated the analysis of various aspects, including countries/regions, institutions, authors, journals and keywords related to the research.

**Results:**

From 2003 to 2023, a total of 1328 articles were retrieved in WOS, and 637 articles were selected by two authors. The most productive institutions are Childrens Hosp Philadelphia, Kocher, ms. Their articles have the highest number of publications and citations. The American journal of sports medicine is the most frequently cited journal for articles on anterior cruciate ligament reconstruction in children and adolescents. The most common keywords used in these articles were “anterior cruciate ligament reconstruction”, “injury, children, adolescent”, and “skeletally immature patients”.

**Conclusions:**

This study provides valuable insights into the research focus of anterior cruciate ligament reconstruction in children and adolescents. In recent years, there has been significant attention paid to areas of “the return to sport, re-repture rate and functional recovery after anterior cruciate ligament reconstruction” in this specific population. These aspects have emerged as key directions for future research in this field.

**Supplementary Information:**

The online version contains supplementary material available at 10.1186/s13018-024-04829-2.

## Introduction

Anterior cruciate ligament (ACL) injury is a common sports injury. In recent years, the incidence of ACL injury in children and adolescents is on the rise [1–4], According to New York State registration data, the ACL reconstruction(ACLR) rate per 100,000 people aged 3 to 20 rose from 17.6 in 1990 to 50.9 in 2009 [5]. Over the past 20 years, the number of ACL breaks in patients aged 6–18 years has increased by at least 2.3% per year, with higher rates in females aged 16 years and males aged 17 years [6], The increase in ACL injuries among children and adolescents may be attributed, in part, to increased participation in physical activities and sports, leading to a higher risk of injury. Additionally, there has been an increased utilization of magnetic resonance imaging(MRI)testing following knee sprains, which allows for better detection and diagnosis of ACL injuries that may have previously gone unnoticed [7]. However, the population of children and adolescents with ACL injury shows an increasing incidence of knee instability, cartilage injury, premature cartilage degeneration, and meniscus injury, and ACL re-rupture and contralateral ACL injury after ACLR are 2–3 times higher in children and adolescents than in adults [8–10]. The current mainstream treatment is arthroscopic anterior cruciate ligament reconstruction [11]. Because the epiphysis of children and adolescents is not closed and is in the stage of growth and development [12–15], the hot spots of return to sport, re-rupture rate, and growth disorder in this population after ACL reconstruction are worthy of our further exploration. Therefore, it is very important to quantitatively analyze the current research hotspots and future research prospects for ACLR in children and adolescents.

Bibliometrics analyzes literature by combining mathematics and statistics. It is a scientific discipline that reveals the law of literature information and literature information management [16] ,Through quantitative analysis of the literature and understanding of the development of this field, we can provide guidance for effective scientific research activities in the future. Therefore, the purpose of this study is to conduct an in-depth study on the reconstruction of anterior cruciate ligament injury in children and adolescents, and to evaluate the current research field, research hotspots and development trends.

## Method

### Data collection

In this study, the Web of Science Core Collection (WoSCC) database was used to search for publications. To avoid bias caused by database updates, the paper search was completed in November, 2023.

**Fig. 1:**
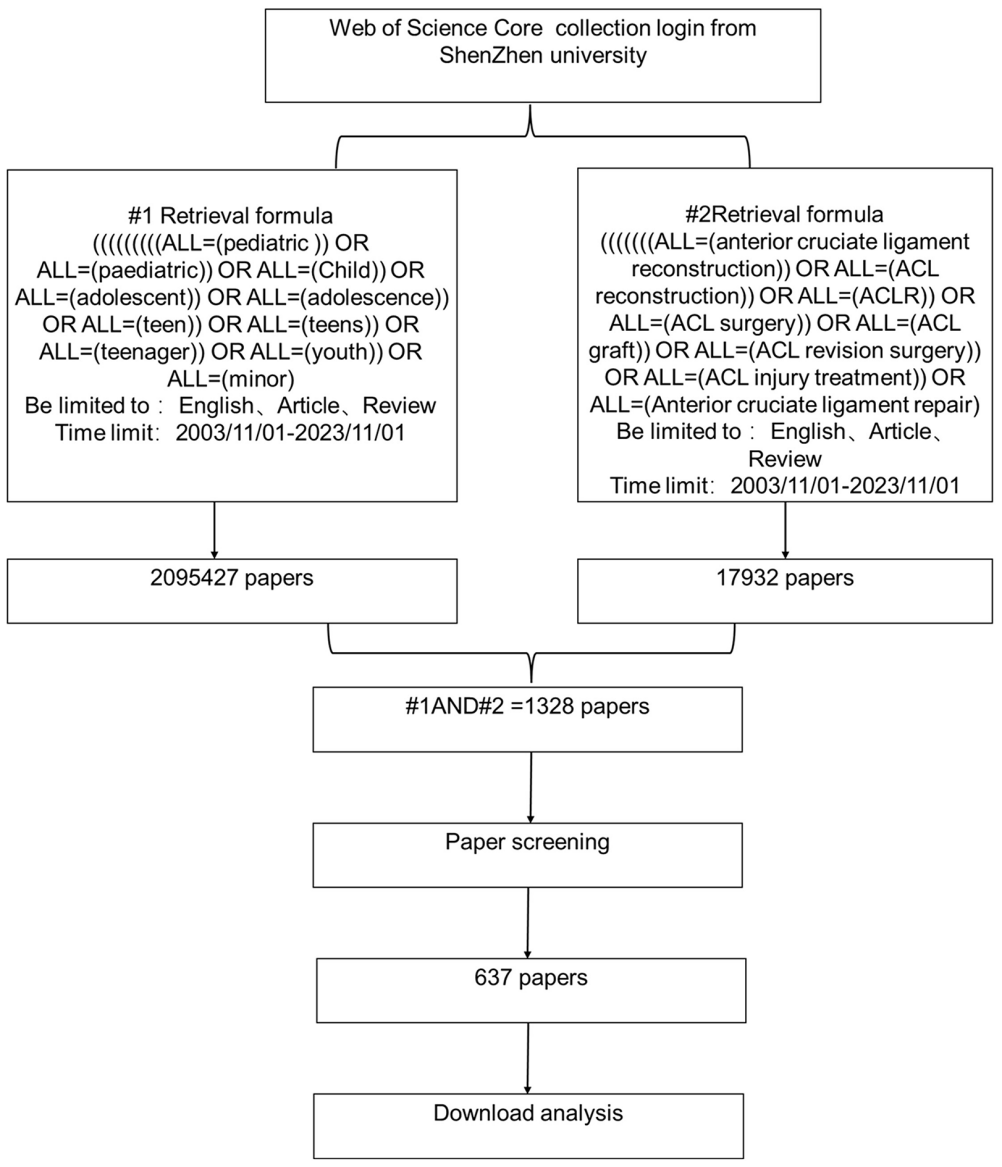
The article is depicted in the flowchart

The flowchart for retrieving the article is shown in Fig. 1. Two different search criteria were used for this study; as shown in Fig. 1, the first (#1) standard targets terms related to the child and adolescent population, while the second (#2) standard focuses on terms related to ACL injuries and their treatment. The standards are grouped together using the AND operator, and the time limit is between November 1, 2003 and November 1, 2023, and the language is limited to English. In addition, the search is limited to articles and reviews.

This comprehensive approach is designed to collect relevant studies on the treatment of ACL injuries in children and adolescents between 2003 and 2023, including those referring to this population using different terms.

### Data screening

Filter the data by two authors and import all the search results into Endnote (X9). First, the repeated studies were filtered, and then the two authors independently assessed the reading of abstracts and titles. If the literature describes the study population as: children or adolescents, the epiphysis is not closed, and the study content is: anterior cruciate ligament injury and reconstruction, then it is included. If a controversial article is selected by two independent authors, a third independent author evaluates the title, abstract, and full text to make a final decision on inclusion.

### Data analysis

VOSviewer 1.6.19, Origin 2022, Pajek64 5.18 and Microsoft Excel 2019 were used for bibliometric analysis and visualization. The VOSviewer is used for co-citation and co-occurrence analysis and the document grid map is created and visualized. Pajek64 5.18 is used to edit the VOSviewer visualization map, making the resulting visual image more intuitive. Use Origin 2022 for visual analysis, including visualization of annual growth trends. The reference index includes the number of papers (n) and the number of citations. When using VOSviewer 1.6.19 for keyword analysis, the parameters are set to the following values: Keyword Repeat Number 7 and Keyword Synonym Substitution, as shown in Table 1.

**Table 1:** Keyword-synonym substitution

Label	Replace by
Acl	Anterior cruciate ligament
Injuries	Injury
Rupture	Injury
Tears	Injury
Adolescents	Adolescent
Skeletally immature	Skeletally immature patients
Acl reconstruction	Anterior cruciate ligament reconstruction
Risk-factors	Risk
Reconstruction	Anterior cruciate ligament reconstruction

## Result

According to the WoSCC database, from 2003 to 2023, 2269 authors from 753 institutions in 41 countries published 637 articles on ACL reconstruction in children and adolescents in 94 journals. Compared to 2003, the number of articles published in 2023 has increased several times, and research on ACLR in children and adolescents has increased steadily over the past 20 years. The increasing trend in the number of papers suggests that ACLR in children and adolescents is attracting more and more attention and interest among researchers.

### Annual growth trend

**Fig. 2:**
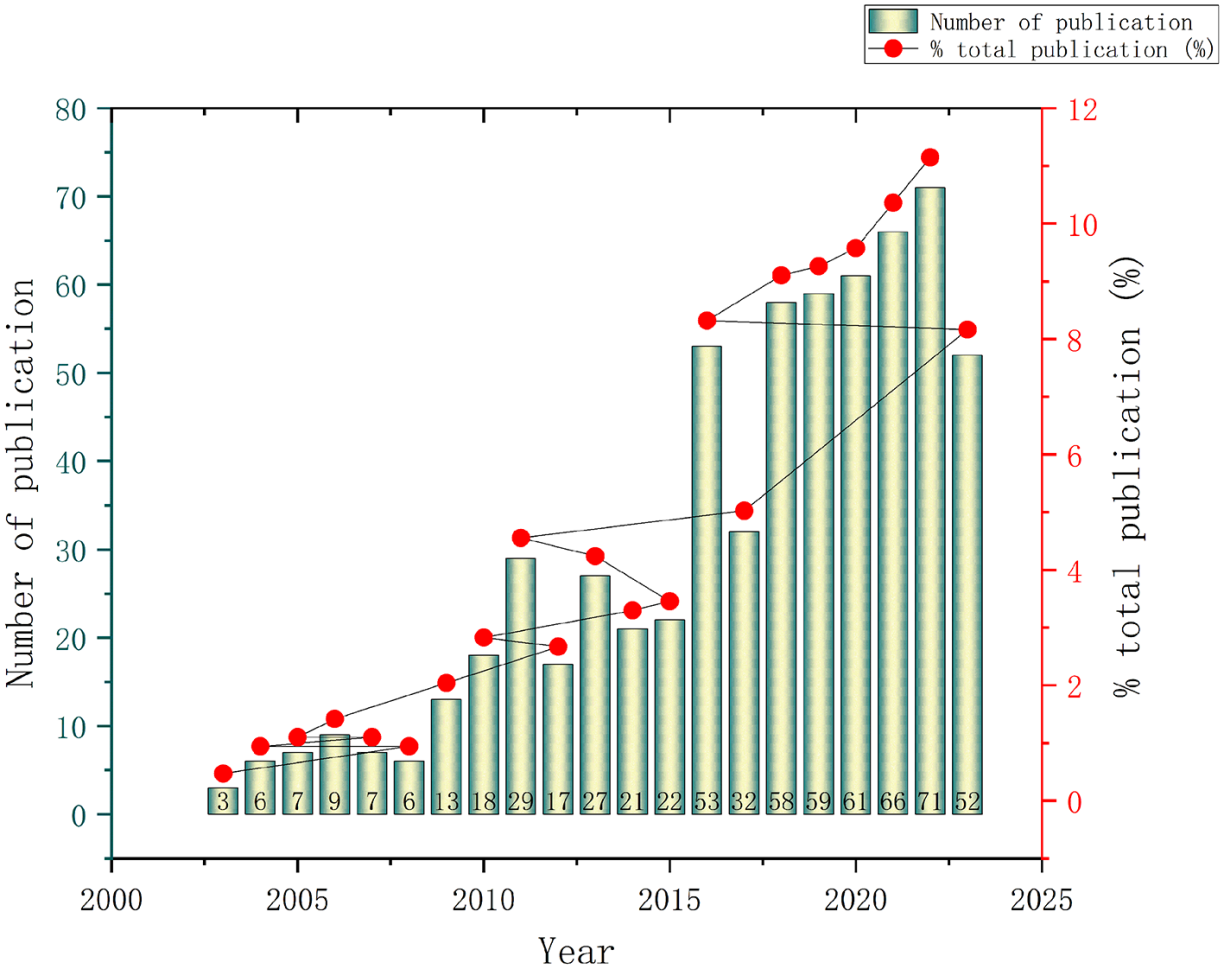
Trend of published papers related to ACL injuries and reconstructions in children and adolescents over the past 20 years

637 papers on ACLR in children and adolescents selected from the WoS database were analyzed. As shown in Fig. 2, the number of papers on ACLR in children and adolescents has fluctuated and increased over the past 20 years.

### Contribution of a country or region

Table 2 shows that over the past 20 years, the 10 countries or regions with the highest number of publications related to ACLR injuries and ACLR in children and adolescents. Six collaboration groups were formed in the 19 most productive countries (≥ 5) (Supplemental Fig. 1). The United States published the most articles (*n* = 434), followed by France (*n* = 40) and Canada (*n* = 36). The United States has the most citations. It is worth noting that although Australia is not in the top three articles (*n* = 26), it ranks second in number of citations, while Canada ranks third in number of citations. There is a slight difference in the number of papers and the number of citations in other countries. In addition, the United States is the country with the highest number of published articles and citations, indicating that the United States is a leader in research on ACL reconstruction in children and adolescents. Finally, Swedish and Danish researchers have published additional papers in recent years (Supplemental Fig. 2) that provide us with some research directions.

**Table 2:** The 10 countries and regions with the highest number of publications related to ACL injuries and reconstructions in children and adolescents in the last 20 years

Rank	Country	Documents	Citations	% of (637)
1	USA	434	11,246	68.13%
2	France	40	798	6.28%
3	Canada	36	802	5.65%
4	Italy	30	650	4.71%
5	Australia	26	1313	4.08%
6	Norway	23	694	3.61%
7	Sweden	21	686	3.30%
8	Japan	20	146	3.14%
9	England	18	633	2.83%
10	Germany	16	507	2.51%

### The contribution of institutions

Table 3 lists the 10 most common institutions in the study for ACLR in children and adolescents over the past 20 years. Philadelphia Children’s Hospital had the largest number of articles (*n* = 59) and citations, followed by New York Special Surgery Hospital (*n* = 50) and Boston Children’s Hospital (*n* = 40). Philadelphia Children’s Hospital and New York Special Surgery Hospital are the largest contributors to research on ACLR in children and adolescents, and their combined output is more than six times that of Cincinnati Children’s Hospital, which has long been one of the top three in the United States. Philadelphia Children’s Hospital ranks first in the number of articles and citations. On the surface, the institution has good academic capabilities. From the top 10 large research institutions, it can be concluded that all of the top 10 institutions are American institutions. Our research shows that there are more facilities in the United States dedicated to reconstructing ACL injuries in children and adolescents. 62 of the most productive institutions (papers ≥ 5) form 4 collaboration clusters. The largest collaborative cluster of research institutions based at the Children’s Hospital of Philadelphia (Supplemental Fig. 3).

**Table 3:** 10 of the most common institutions investigating ACL injuries and reconstructions in children and adolescents over the past 20 years

	Organization	Documents	Citations	% of (637)
1	Childrens hosp philadelphia	59	1425	9.26%
2	Hosp special surg	50	1075	7.85%
3	Boston childrens hosp	40	531	6.28%
4	Harvard med sch	30	629	4.71%
5	Univ penn	20	373	3.14%
6	Connecticut childrens med ctr	18	287	2.83%
7	Univ cincinnati	18	1295	2.83%
8	Rady childrens hosp	17	171	2.67%
9	cincinnati childrens hosp med Ctr	16	1064	2.51%
10	Univ calif san diego	16	297	2.51%

### Author analysis

Table 4 lists the 10 authors with the most published articles. The 10 authors published a total of 227 articles, accounting for 35.64% of the total. Ganley, from the Children’s Hospital of Philadelphia in Pennsylvania, USA, ranked first in the study of anterior cruciate ligament injury reconstruction in children and adolescents, followed by Kocher from Boston Children’s Hospital, Department of Orthopedic Sports Medicine, Harvard Medical School, Boston, Massachusetts, USA. So if you want to continue following the development of ACLR in children and adolescents, you can follow these authors: Ganley, Theodore J. And Kocher, Mininder s. The number of cited papers is very high, which shows that their work has attracted the attention of researchers. However, through VOSviewer analysis, we can see that the collaboration between authors of collaborative clusters is not sufficient (Supplemental Fig. 4).

**Table 4:** 10 authors with the most published articles

Rank	Author	Documents	Citations	% of (637)
1	Ganley, Theodore j.	47	1273	7.38%
2	Kocher, Mininder s.	33	811	5.18%
3	Fabricant, Peter d.	28	625	4.40%
4	Green, Daniel w.	23	690	3.61%
5	Shea, Kevin g.	21	242	3.30%
6	Engebretsen, Lars	18	580	2.83%
7	Micheli, Lyle j	15	479	2.35%
8	Moksnes, Havard	14	539	2.20%
9	Milewski, Matthew d.	14	210	2.20%
10	Patel, Neeraj m.	14	135	2.20%

In “Trends in Pediatric ACLR From the PHIS Database” the Kocher team’s most widely cited article, Kocher et al. analyzed data from pediatric hospitals in the United States from 2004 to 2014 and found the number of ACL reconstructions increased 5.7-fold, whereas orthopaedic surgeries increased 1.7-fold; there was a 2.8-fold increase in ACL reconstructions relative to total pediatric orthopaedic surgeries. This is significantly higher than the overall orthopedic surgery growth rate (1.7 times). The annual rate of ACL reconstruction was 32.4 per 1000 orthopedic surgeries, a nearly three-fold increase compared to the total number of orthopedic surgeries.

Moreover, 80% of the top 10 authors hail from the United States, a country renowned for its plethora of exceptional researchers dedicated to the study of ACL injury and reconstruction in children and adolescents. This underscores the profound importance these researchers place on advancing knowledge in this field.

### Magazine analysis

A total of 94 journals have published articles on ACL injuries and reconstructions in children and adolescents. The top 10 journals published a total of 412 articles, accounting for 64.68% of the total, as shown in Table 5. Among them, the American Journal of Sports Medicine (99) was the journal with the largest number of published articles, followed by Orthopedic Journal of Sports Medicine (75) and Journal of Pediatric Orthopedics (71). The reason why these journals are in the top 10 is, firstly, because the research topics they cover are most relevant to the reconstruction of ACL injuries in children and adolescents, and relevant researchers are often happy to submit papers to these journals. Secondly, these journals are the most influential professional journals in the field, and researchers can effectively improve their academic level and research ability by reading the papers published in these journals.

**Table 5:** The ten journals with the highest number of published articles

Rank	Source	Documents	Citations	JCR partition	Influence factor	Classification	State
1	American journal of sports medicine	99	5052	Q1	4.8	Sports medicine	UNITED STATES
2	Orthopaedic journal of sports medicine	75	625	Q2	2.6	Orthopaedics / sports medicine	United States
3	Journal of pediatric orthopaedics	71	1620	Q3	1.7	Pediatrics / orthopaedics	UNITED STATES
4	Knee surgery sports traumatology arthroscopy	64	1679	Q1	3.8	Orthopaedics / sports medicine	GERMANY
5	Arthroscopy-the journal of arthroscopic and related surgery	36	1160	Q1	4.7	Orthopaedics / sports medicine	UNITED STATES
6	Clinics in sports medicine	14	241	Q3	2	Sports medicine	UNITED STATES
7	Journal of athletic training	14	341	Q2	3.3	Sports medicine	UNITED STATES
8	Knee	14	207	Q3	1.9	Sports medicine / orthopaedics	NETHERLANDS
9	Orthopaedics & traumatology-surgery & research	13	120	Q2	2.3	Surgery / orthopaedics	FRANCE
10	Journal of bone and joint surgery-american volume	12	764	Q1	5.3	Surgery / orthopaedics	UNITED STATES

### Analysis of papers cited by high frequency

Table 6 shows the 10 most frequently cited papers on ACL injury and reconstruction in children and adolescents over the past 20 years (2 reviews and 8 research articles). These articles were primarily published between 2004 and 2016, and they summarized and analyzed the epidemiology, etiology, treatment and prognosis of ACL injury and reconstruction in children and adolescents. An increase in the rates of ACL reconstruction in individuals with immature skeletal development was noted [5]. Firstly, cruciate ligament injuries are common among young soccer players, particularly female athletes. Secondly, studies have shown that the risk of re-injury following ACLR is closely related to age and activity level, particularly in athletes under 25 years of age returning to high-risk sports [17]. Furthermore, the management of ACL injuries in patients with immature bones remains controversial, but techniques such as epiphyseal preservation and combined reconstruction have shown favorable functional outcomes and low revision rates in preadolescent children [18]. In addition, the literature also mentions the significant increase in knee abduction angle [18] observed in female athletes during the rapid growth phase of adolescence [18–20], which may be associated with an increased risk of ACL injury. Finally, the literature indicates that young athletes who return to sports after an ACLR have higher rates of secondary injuries, with a risk 30 to 40 times higher than the initial ACL injury. Therefore, some measures must be taken to reduce secondary injury in this high-risk population [17].

**Table 6:** Shows the ten most frequently cited papers on ACL injuries and reconstructions in children and adolescents over the past 20 years

Rank	Document	Citations
1	Wiggins (2016)	605
2	Dodwell (2014)	255
3	Padua (2015)	239
4	Lawrence (2011)	209
5	Quatman (2006)	200
6	Shea (2004)	199
7	Maffulli (2010)	196
8	Ford (2010)	187
9	Kocher (2005)	187
10	Anderson (2015)	184

### Keyword analysis

**Fig. 3:**
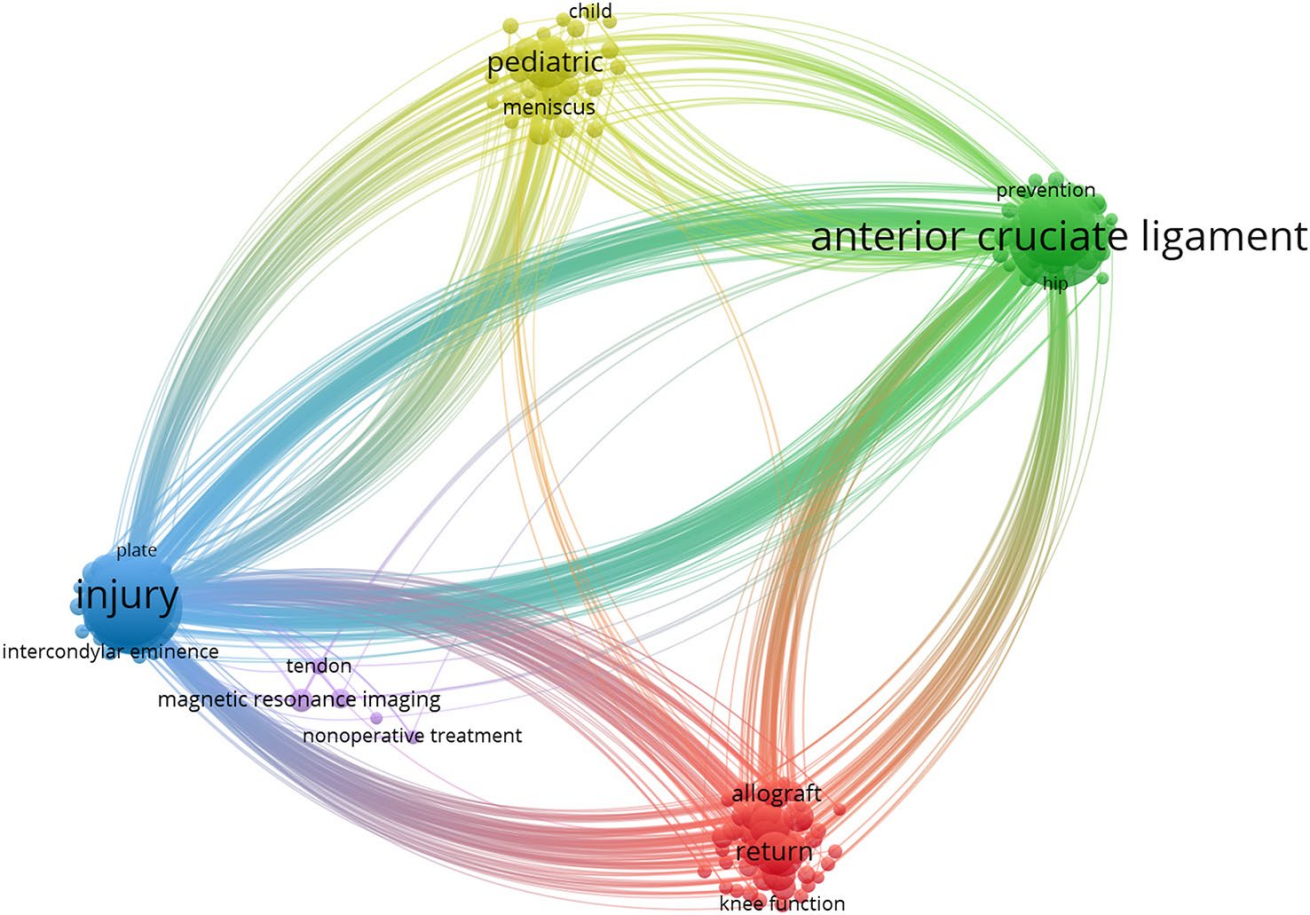
Keyword co-occurrence network

Using VOSviewer, we analyzed 1721 keywords found in titles and abstracts of 637 articles. From this analysis, we identified 5 clusters represented by different colorsn as shown in Fig. 3: red, green, yellow, purple and blue. These clusters were formed by 162 keywords that occurred more than 7 times. Cluster 1 (red) primarily focused on the prognosis and return to sport following ACL reconstruction in children and adolescents. Cluster 2 (green) primarily investigated the risk factors associated with ACL injury in children and adolescents. Cluster 3 (blue) mainly focused on the characteristics of ACL injury and reconstruction in children and adolescents, including open epiphysis, growth and development stage, etc. Cluster 4 (yellow) mainly showed a series of complications of ACL injury Cruciate ligament in children and adolescents. Cluster 5 (purple) focused on the diagnosis of ACL injury. The five most commonly classified were “anterior cruciate ligament injury,” “anterior cruciate ligament reconstruction,” “children and adolescents,” and “skeletally immature patients”. In addition, the keyword time chart (Supplemental Fig. 6) provides valuable information about the publication trends. In this chart, the color yellow represents the newest keywords associated with the average publication, while blue represents the oldest keyword. By analyzing the chart, we observed that the most publications in this field have focused on several key areas. These included “return to sport”, “re-rupture”, “outcome” and “functional prediction after ACL reconstruction”. These results showed that the concept of “function first” was becoming increasingly popular and researchers in related fields were paying more and more attention to the reconstructed functions of the ACL.

## Discussion

This study visually analyzed the research results related to ACL injury reconstruction in children and adolescents to summarize the current situation and development trend of research in this field. Through the comprehensive evaluation of the number of articles, countries and institutions, major researchers and research hotspots, the following key findings were made: Firstly, the incidence of ACL damage in children and adolescents increases with increase participation in physical activities [2–4, 8, 21–24]. Related studies have shown that the high incidence of ACL injuries in children and adolescents is on one hand due to the increased participation in physical activity and the lack of sports experience or conditioned reflexes of children and adolescents [25], and on the other hand, the improvement of imaging and diagnostic technology [26–30]. Arthroscopic ACL reconstruction is the gold standard for the treatment of ACL rupture in children and adolescents [11]. However, the re-rupture rate of ACL reconstruction in children and adolescents is higher than that in adults [31–35]. The reason may be due to early return to sports [25, 36]. The compliance of children and adolescents is generally poor. Consequently, there has been an increase in research output in this field, indicating the growing attention and efforts of researchers and clinicians to address this increasingly significant problem. Secondly, the United States maintains a leading position in the field of ACL injury reconstruction in children and adolescents. This can be attributed to several factors, including its advantages in the allocation of medical resources, research funding, and long-term expertise in the field of sports medicine. As shown in Supplemental Fig. 2, the rapid growth of research results in countries such as Denmark and Sweden shows that Europe is gradually exerting more influence in this field. In terms of research institutions, Philadelphia Children’s Hospital, New York Special surgery Hospital and Boston Children’s Hospital in the United States have made significant contributions to the study of ACL injury reconstruction in children and adolescents. The excellent performance of these institutions not only reflects their leading position in sports medicine research, but also emphasizes the importance of high-level research in promoting medical quality. The research works of Professor Theodore J. Ganley and Professor Mininder S. Kocher have had a significant and far-reaching impact on the field of ACL injury and reconstruction in children and adolescents. Their contributions have not only advanced the accumulation of professional knowledge, but also provided valuable guidance for clinical practice.

In terms of research hotspot, significant keywords such as “return to sport”, “re-rupture”, “outcome” and “functional prediction after ACL reconstruction” have emerged as key areas of focus in current studies. These keywords highlight the main direction that researchers are exploring, and their findings are consistently being published in authoritative journals. These research hotspots reflect the dedication of sports medicine researchers to enhancing the success rate of ACL reconstruction surgery, optimizing postoperative rehabilitation, and maximizing the recovery of patients’ motor function.

### Advantages and limitations

Based on bibliometric analysis and literature visualization, we can gain a better understanding of the research trend and tendencies of ACL injuries and reconstructions in children and adolescents. By analyzing the number of citations and articles, we can first objectively assess the research results, the influence of those results and the overall quality of research institutions. Secondly, we can visually analyze the literature, examine the relationship between the literature, and find some directions with potential research value. Offer researchers new ideas and directions.

However, this study has some limitations. Firstly, the literature from the Web of Science core database only contains relevant articles or reviews in English. Secondly, VOSviewer 1.6.19 and Excel Office 2019 of bibliometric analysis cannot analyze the full text of articles and some information may be ignored. Finally, some recently published articles may be ignored due to their low citation frequency and the impact may be underestimated. Some recently published articles may have better chance of accumulating citations if published earlier.

### Conclusion and prospect

In recent years, there has been increasing interest in studying the “function, return to sport, re-rupture rate and functional recovery of ACL reconstruction in children and adolescents. This widespread attention has provided a clear direction for future research in this field. When examining the number of articles published on the reconstruction of ACL injuries in children and adolescents over the past 20 years, there has been some fluctuation, but the overall trend indicates an increase in research output. This suggests a growing recognition of the importance of studying ACL injuries and reconstructions in this specific population. The United States and some European countries are at the forefront of research in this field. Relevant researchers can track the latest research results to guide the prevention and treatment of ACL injuries in children and adolescents.

In the future, in order to further improve the outcomes of ACL reconstruction surgery and the rehabilitation quality of patients, the research should focus on the innovation of surgical techniques, the optimization of postoperative rehabilitation programs and strategies to prevent re-rupture.

### Practical implications

1. This study reports that the rate of ACL re-rupture after reconstruction in children and adolescents is higher than that in adults. When this group returns to sports, it is necessary to comprehensively evaluate the sports ability, strengthen the monitoring of sports indicators, guide the return to sports, and pay attention to sports protection.
2. The incidence of ACL injury in children and adolescents is on the rise. It is necessary to improve the sports skills of children and adolescents, and strengthen the early screening and diagnosis of ACL injury.
3. The treatment and prognosis of ACL reconstruction in children and adolescents are different from those in adults. although there are many studies on ACL reconstruction, they focus on adults, and there are great physiological and psychological differences between adults and children. it is of little significance to guide the reconstruction of children and adolescents, so we should consider making injury and disease prevention strategies for children and adolescents.

### Electronic supplementary material

Below is the link to the electronic supplementary material.

Supplementary Material 1

## Data Availability

No datasets were generated or analysed during the current study.
