# Supplementary material for: Global overview of anterior cruciate ligament reconstruction in children and adolescents over the past 20 years: a bibliometric analysis
Source: J Orthop Surg Res. 2024 Jun 13;19:350. doi: 10.1186/s13018-024-04829-2 (PMC11170893; doi:10.1186/s13018-024-04829-2)
Supplement: Supplementary file 1 — Supplementary Material 1 [file 13018_2024_4829_MOESM1_ESM.docx]

**Supplemental figure**


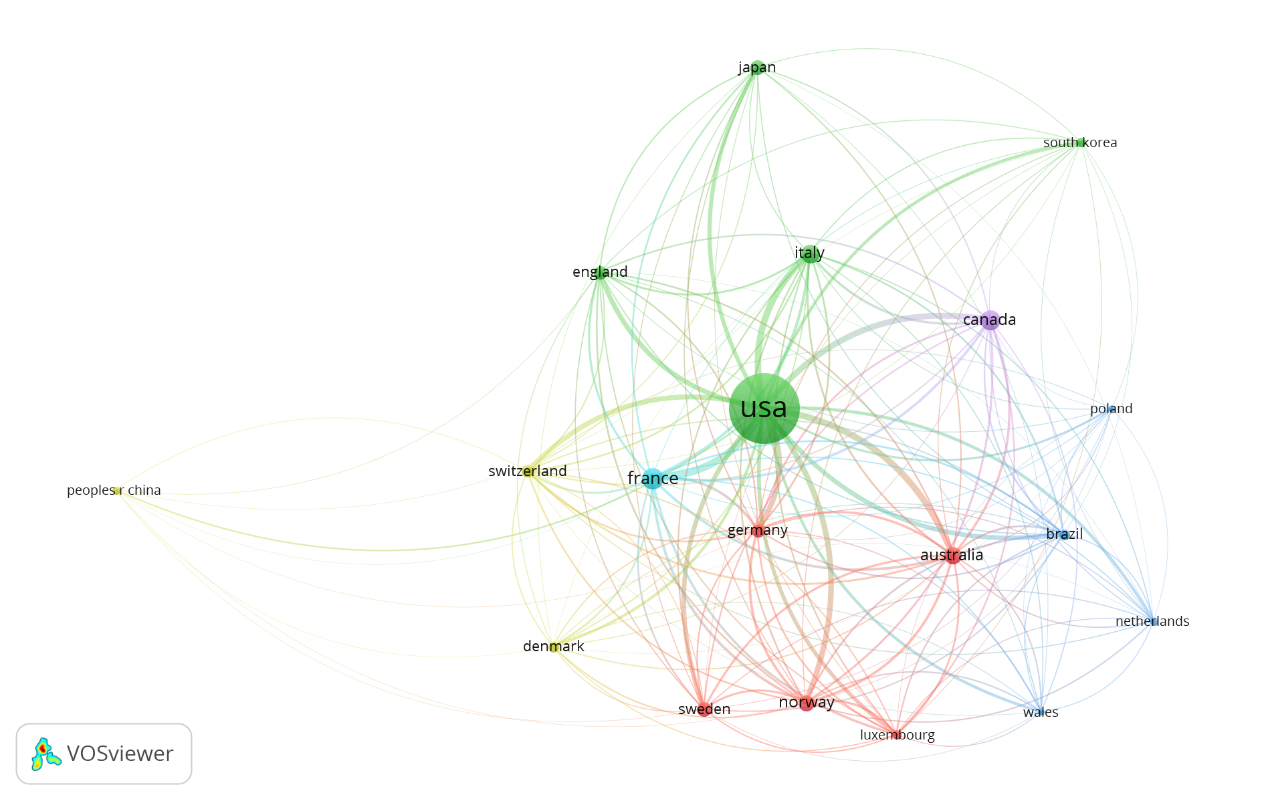


**Supplemental Fig. 1.** Co-occurrence Network Map of Countries and Regions.


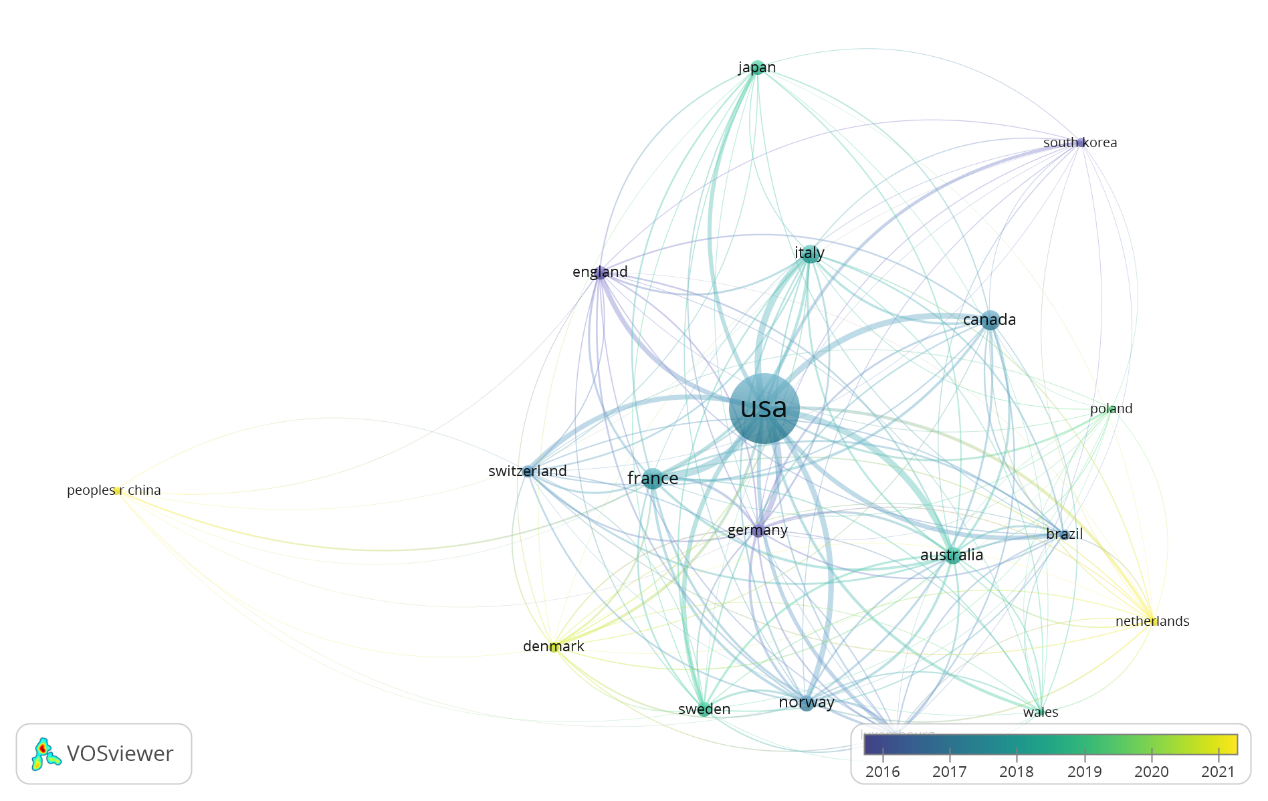


**Supplemental Fig. 2**. Time Chart of Co-occurrence Network in Countries and Regions.


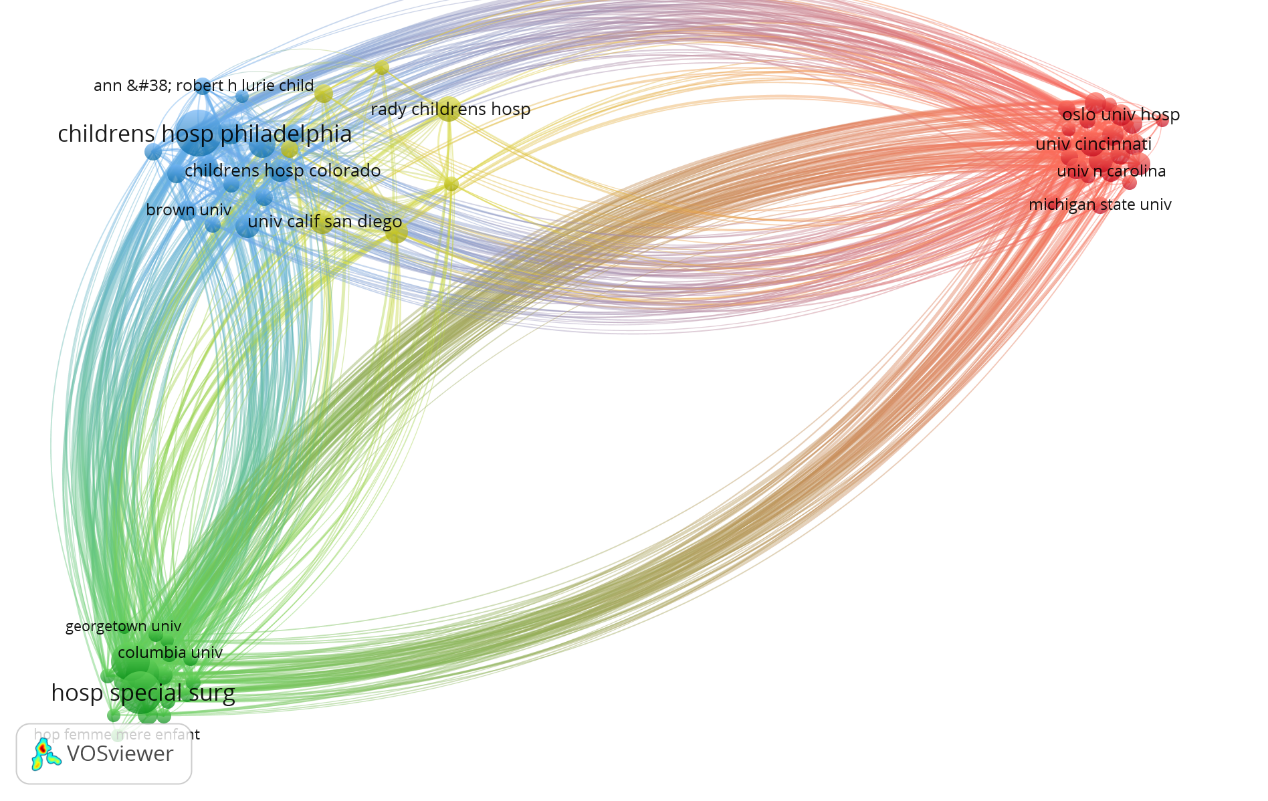


**Supplemental Fig. 3 − 1**.Institutional Co-occurrence Network Diagram.


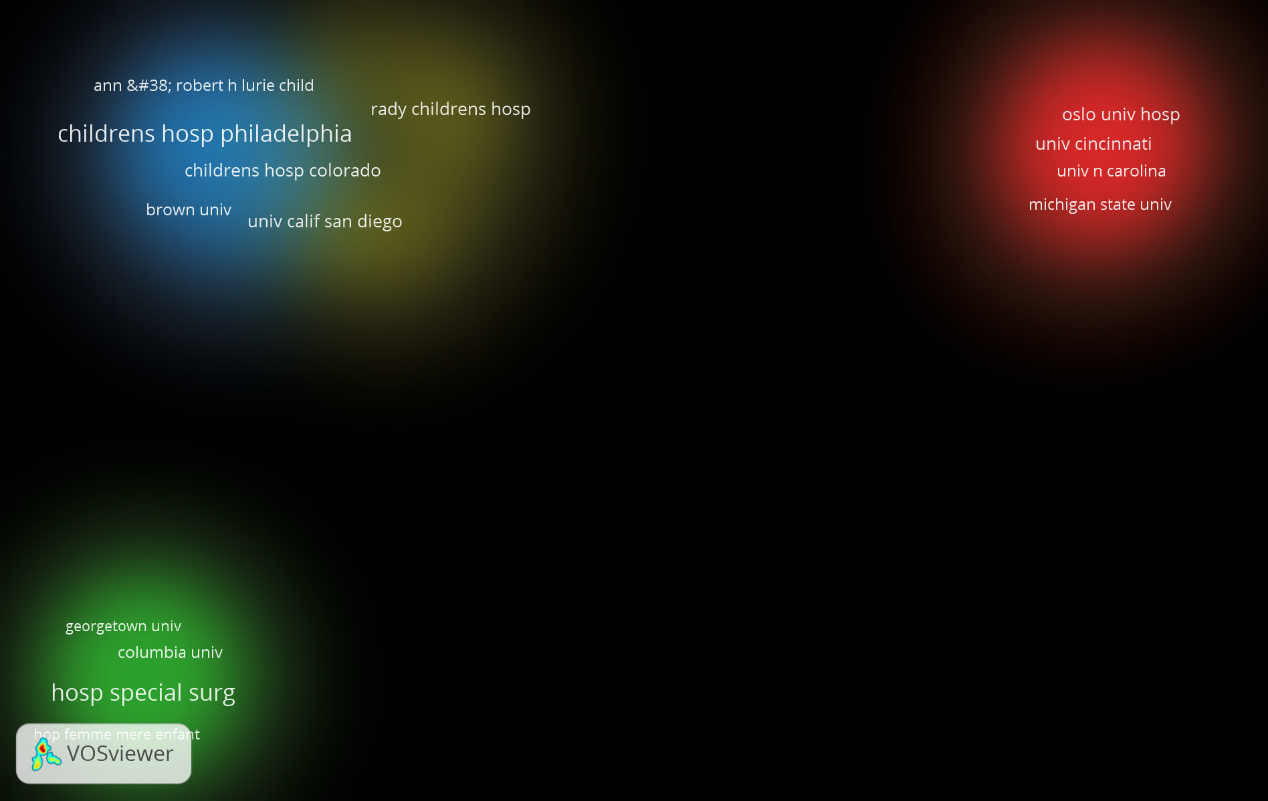


**Supplemental Fig. 3 − 2.** Institutional Co-occurrence Network.


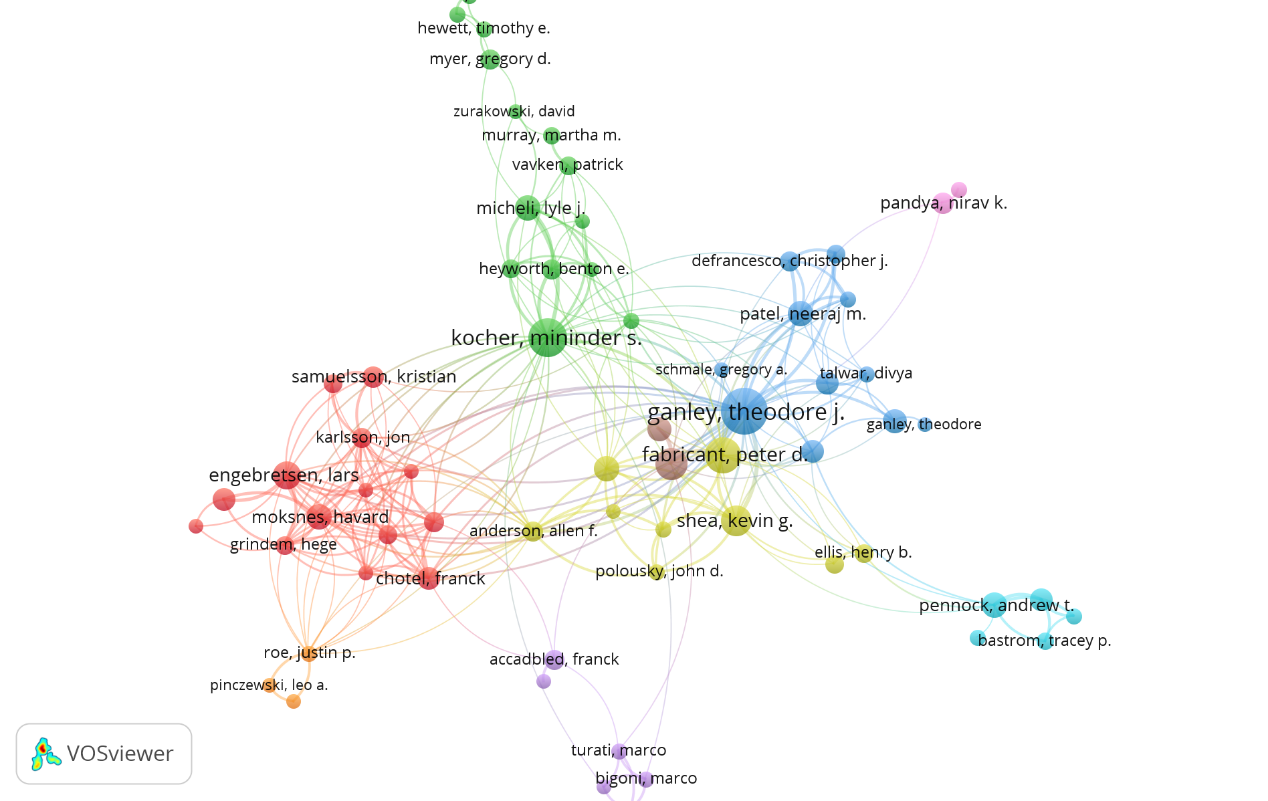


**Supplemental Fig. 4 − 1.**the author's co-occurrence network diagram.


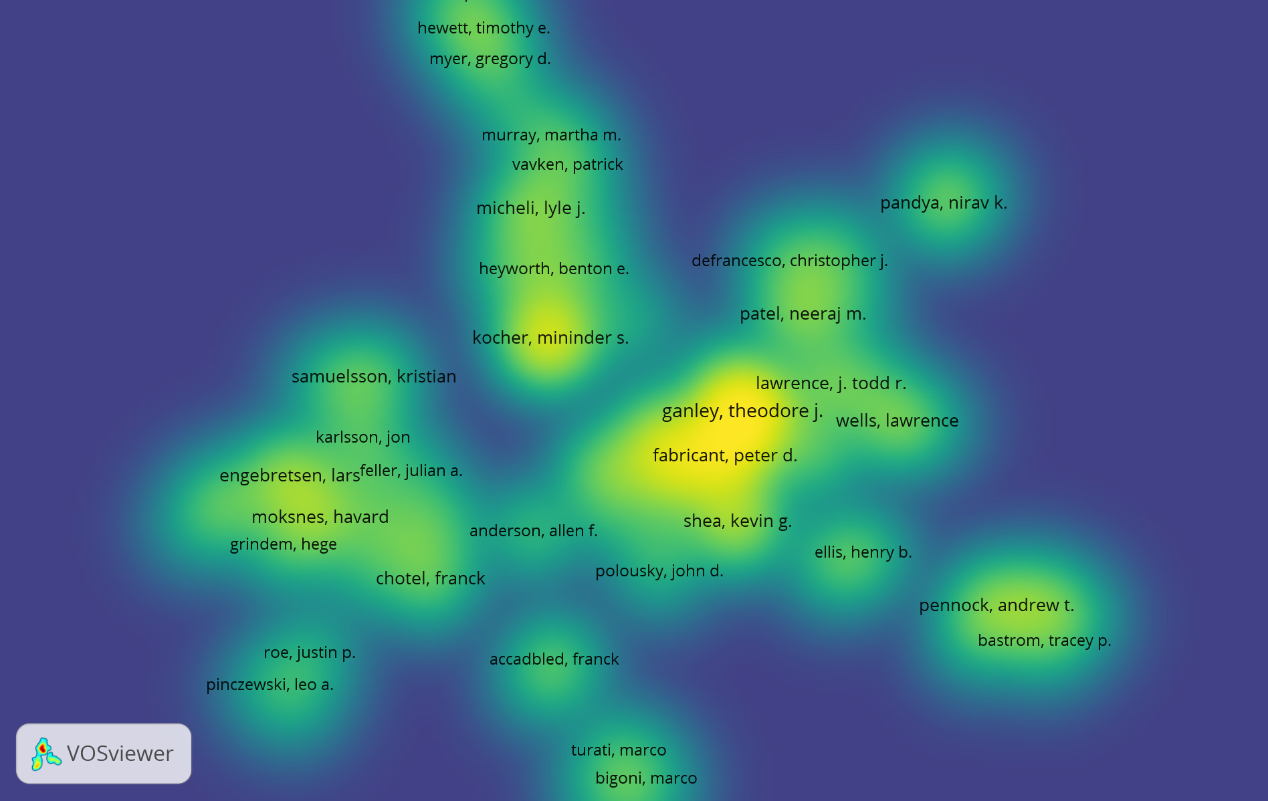


**Supplemental Fig. 4 − 2**.The co-occurrence network diagram of the author.


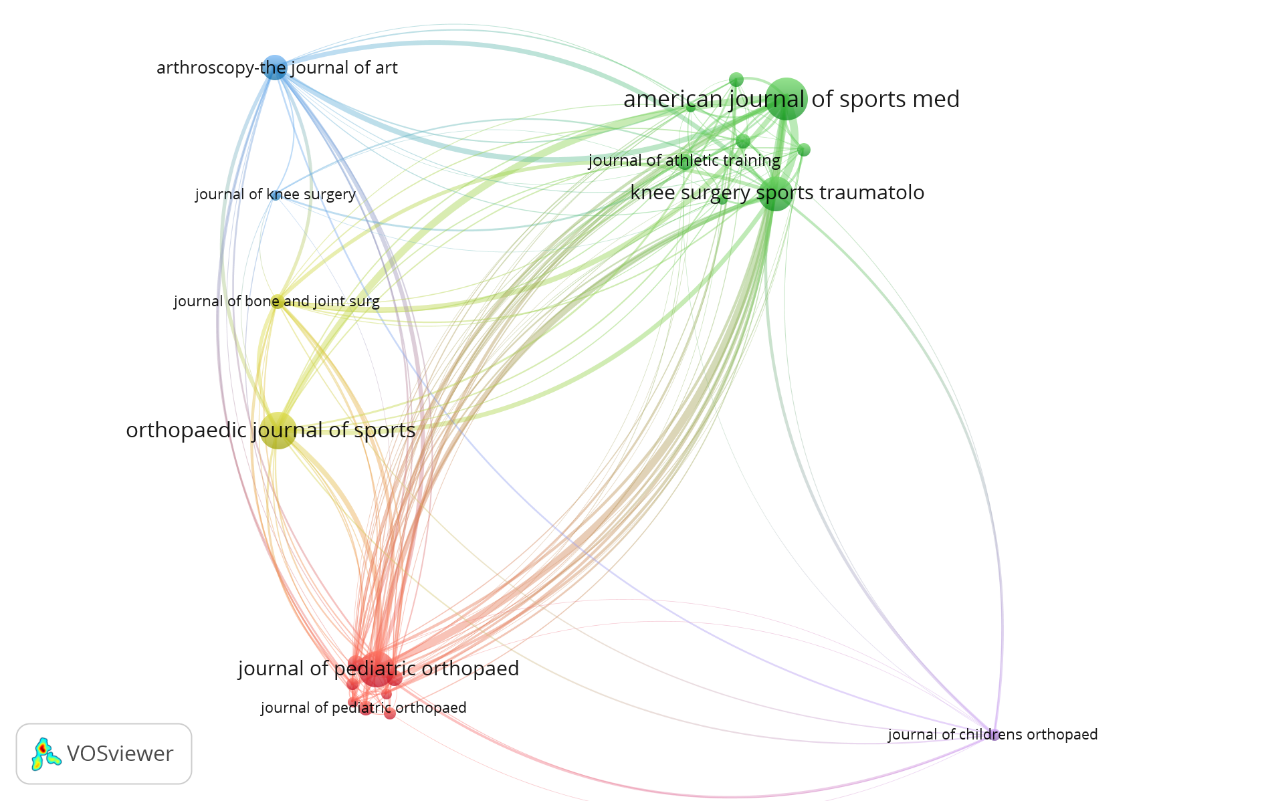


**Supplemental Fig. 5 − 1.**Periodical Co-occurrence Network Diagram.


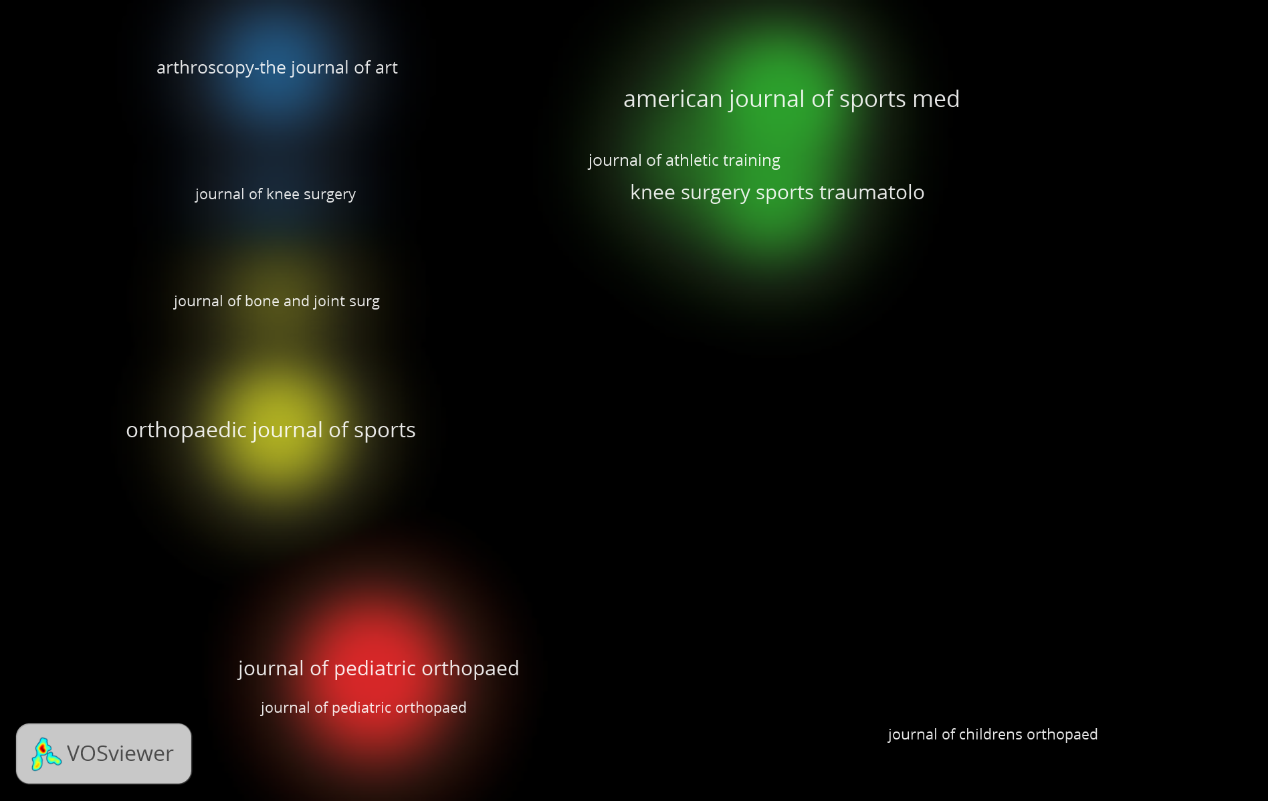


**Supplemental Fig. 5 − 2**.Periodical Co-occurrence Network Diagram.


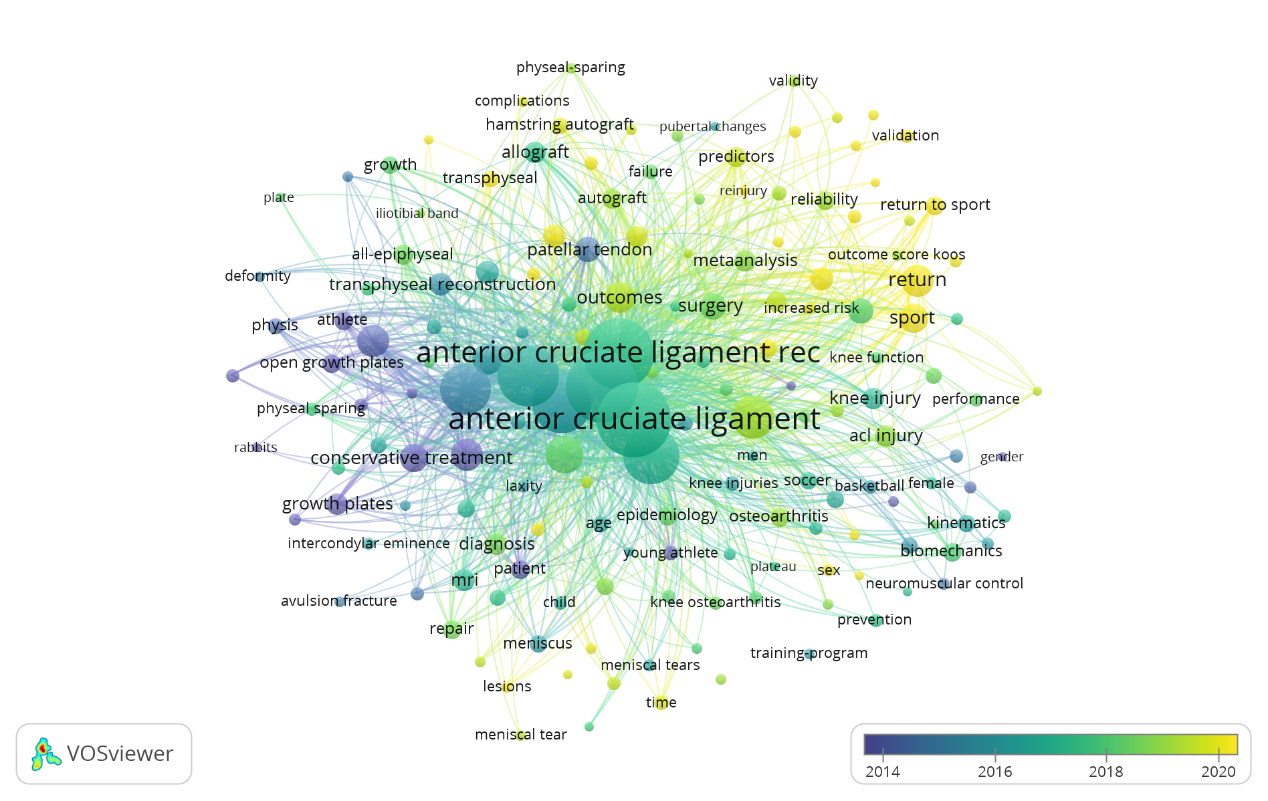


**Supplemental Fig. 6 − 1.**Keyword co-occurrence time network diagram.


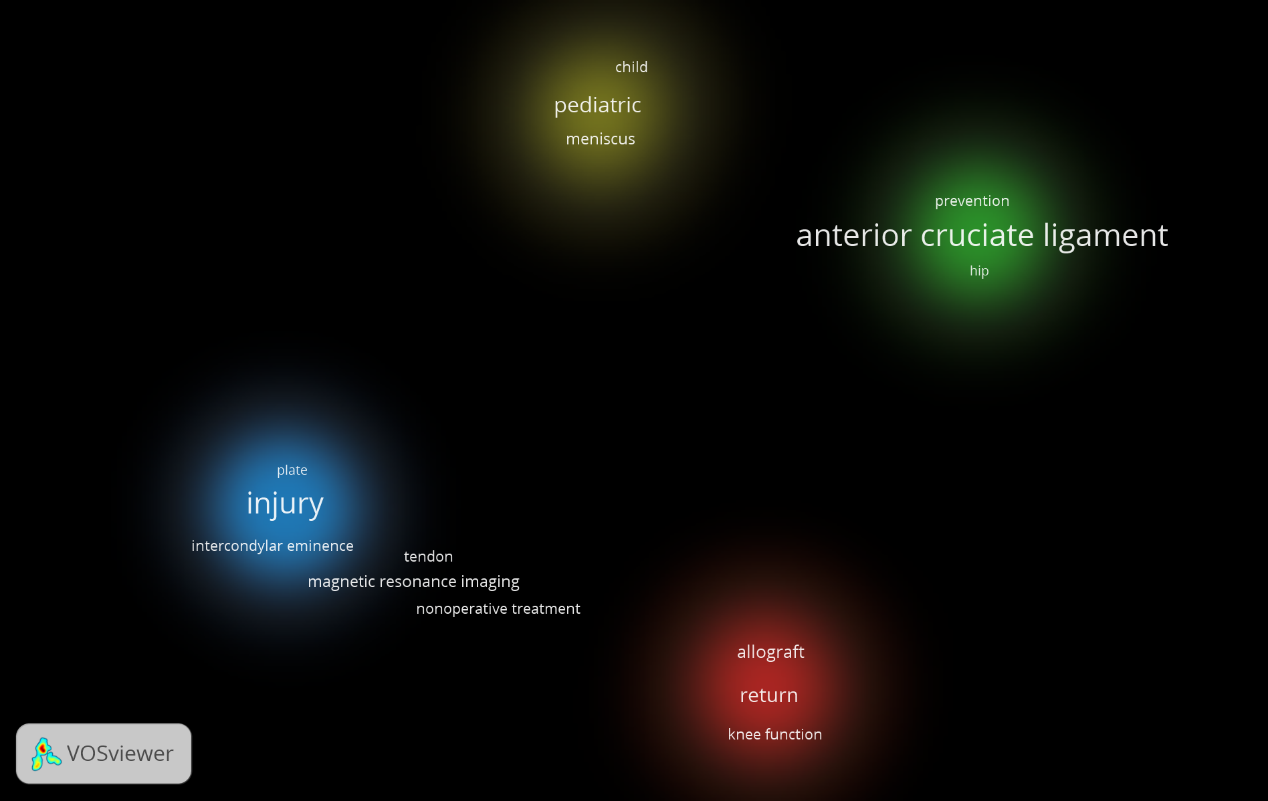


**Supplemental Fig. 6 − 2.** Keyword Co-occurrence Network Diagram.
